# High serum IgA/C3 ratio better predicts a diagnosis of IgA nephropathy among primary glomerular nephropathy patients with proteinuria ≤ 1 g/d: an observational cross-sectional study

**DOI:** 10.1186/s12882-019-1331-0

**Published:** 2019-04-30

**Authors:** Wen-yu Gong, Man Liu, Dan Luo, Fan-na Liu, Liang-hong Yin, Yuan-qing Li, Jun Zhang, Hui Peng

**Affiliations:** 1 Division of Nephrology, Department of medicine, the First Affiliated Hospital, Jinan University, Guangzhou, 510630 Guangdong China; 2 Division of Gastroenterology and Hepatology, The First Affiliated Hospital of Sun Yat-sen University, Guangzhou, Guangdong China; 3 Division of Nephrology, Department of medicine, Third Affiliated Hospital of Sun Yat-Sen University, Guangzhou, 510630 Guangdong China

**Keywords:** Immunoglobulin a nephropathy, Serum immunoglobulin a/C3 ratio, Diagnosis

## Abstract

**Background:**

The serum immunoglobulin A (IgA)/C3 ratio is considered to be an effective predictor of IgA nephropathy (IgAN). This study sought to explore the diagnostic value of the IgA/C3 ratio in IgAN among primary glomerular nephropathy patients in China.

**Methods:**

We recruited 1095 biopsy-diagnosed primary glomerular nephropathy patients, including 757 IgAN patients and 338 non-IgAN patients. Patient demographics, serum immunological indices, and other clinical examinations were measured. IgAN cases were propensity score matched (PSM) to non-IgAN cases on the logit of the propensity score using nearest neighbor matching in a 1:1 fashion, with a caliper of 0.02 with no replacements, according to age, gender, BMI, proteinuria level, and estimated glomerular filtration rate (eGFR).

**Results:**

We found that in both the full cohort and PSM cohort, the IgA/C3 ratio in the IgAN group was significantly higher than that of the non-IgAN group. The same results were also obtained with stratification by different levels of proteinuria and renal function. In the PSM cohort, there was no difference in IgA/C3 ratio in patients with IgAN between different proteinuria groups and different chronic kidney disease (CKD) groups. The area under the ROC curve (AUROC) of the IgA/C3 ratio in distinguishing IgAN among primary glomerular disease was 0.767 in the full cohort, and 0.734 in the PSM cohort. The highest AUROC of the IgA/C3 ratio was in the ≤1 g/d proteinuria group (0.801 in the full cohort, and 0.803 in the PSM cohort); however, there was no difference between all CKD groups. Meanwhile, the diagnostic accordance rate for the diagnosis of IgAN among all patients with an IgA/C3 ratio > 3.5304 was as high as 92.02% in the full cohort. IgAN was independently correlated with IgA/C3 ratio in the full cohort by multivariate logistic regression analysis.

**Conclusions:**

The present study provides clear evidence that the IgA/C3 ratio is an effective predictor of IgA diagnosis, especially in patients with proteinuria ≤1 g/d. In order to study the effectiveness of this biomarker, and to determine a standardized cut-off value, additional multicenter large-scale studies are needed.

**Electronic supplementary material:**

The online version of this article (10.1186/s12882-019-1331-0) contains supplementary material, which is available to authorized users.

## Background

Immunoglobulin A nephropathy (IgAN), which was initially described by Berger and Hinglais in 1968 [1], is the most common type of primary glomerulonephritis worldwide [2–4], especially in the Asia-Pacific region [2, 5, 6]. IgAN accounts for more than 20% of glomerular diseases [5, 6]. An epidemiological survey showed that approximately 45% of the cases of primary glomerulonephritis in China were IgAN [6]. IgAN is diagnosed by renal biopsy with immunofluorescence staining or immunohistochemistry, and is histologically characterized by the presence of polymeric IgA deposits in glomerular mesangial areas [1, 7]. The clinical presentation of IgAN is highly variable between individuals, and ranges from a subclinical disease with isolated microscopic hematuria to severe end-stage renal disease (ESRD). The stereotypical IgAN patient is a young adult with asymptomatic microscopic hematuria or episodic macroscopic hematuria occurring during an upper respiratory tract infection. IgAN was previously considered to be a benign condition, but recent studies have demonstrated that up to 25–40% of patients progress to uremia within 10–20 years after the diagnostic biopsy [6, 8–10].

Until present, the diagnosis of IgAN relies on revealing IgA as the dominant or codominant immunoglobulin in the glomerular mesangium by kidney biopsy [1]. However, the pathogenesis of IgAN remains unclear. According to recent studies, IgAN is thought to be autoimmune in nature. Suzuki et al. put forward a four-hit hypothesis, in which the galactose-deficient IgA1 (GdIgA1) molecule plays a key pathogenic role [11, 12]. Although renal biopsy remains the gold standard for IgAN diagnosis, recent efforts have sought to find new biomarkers that can predict IgAN without the need for biopsy [13–15]. Previous studies have shown that IgA deposition in the mesangial area is necessary for IgAN progression and accompanying C3 deposition. However, the correlation between the changes in serum complement levels and diagnosis remains unclear [12, 16]. Some researchers have reported that IgAN patients usually have higher serum IgA and GdIgA1 levels [17, 18], alongside normal-to-low serum C3 levels, compared to those of patients with other types of primary glomerulonephritis [19, 20]. In 2000, Yasuhiko T et al. reported that patients with higher serum IgA/C3 ratios were more likely to be diagnosed with IgAN [13]. Since then, several studies have sought to evaluate whether the IgA/C3 ratio can serve as a useful clinical indicator to predict the diagnosis and prognosis of IgAN [13–15, 20–22]. However, most of these studies focused on a Japanese patient population and had small sample sizes. Thus, these findings require replication in other patient populations and larger sample sizes to confirm the validity of the association, and to account for potential ethnic differences. Moreover, previous studies have not reported whether the IgA/C3 ratio is of similar value when diagnosing IgAN by proteinuria levels or renal function.

In non-randomized studies, good study methodology not only effectively evaluates the clinical program, but also minimizes confounding variables. In statistical analyses, propensity score matching (PSM) between treatment groups is a practical method to reduce confounding bias, particularly in evaluating comparative effectiveness of two or more variables [23, 24]. Unlike stratification or regression, the new PSM cohort is in equilibrium with the measured covariates during the PSM process, and is also in “clinical equipoise”. In other words, in this selected cohort, every patient should be a reasonable candidate for every treatment being studied [24–26]. Increasing attention is being paid to the use of PSM as a statistical method in clinical studies.

The purpose of this study was to evaluate whether a high serum IgA/C3 ratio, the suggested novel biomarker, could be used as a diagnostic criterion for IgAN in a large Chinese patient population, using PSM methods.

## Methods

### Study population

Between January 2001 and December 2017, 813 IgAN patients, confirmed by renal biopsy at the third affiliated hospital of Sun Yat-Sen University, were recruited as the cohort for this cross-sectional study. Additionally, 264 patients with other forms of primary glomerular nephropathy (non-IgAN group) were recruited between January 2012 and December 2017. Also included were 137 IgAN patients and 146 non-IgAN patients from the First Affiliated Hospital of Jinan University between January 2012 and December 2017. All renal biopsy diagnoses were performed by professional pathologists. The exclusion criteria were as follows: 1) undergoing treatment with corticosteroids, immunosuppressants, or bacteriotoxic drugs; 2) secondary causes of renal diseases such as systemic lupus erythematosus (SLE), Henoch–Schonlein purpura, malignant tumors, hepatic diseases, lymphoma, or other systemic diseases; 3) acute interstitial nephritis; 4) acute changes in the estimated glomerular filtration rate (eGFR) > 30% in the previous 3 months; 5) patients aged < 18 years; 6) pregnancy; 7) acquired immunodeficiency syndrome or other autoimmune diseases; 8) inability to communicate and comply with all of the study requirements; 9) a deficiency of clinical data; and 10) patients undergoing maintenance dialysis.

**Fig. 1:**
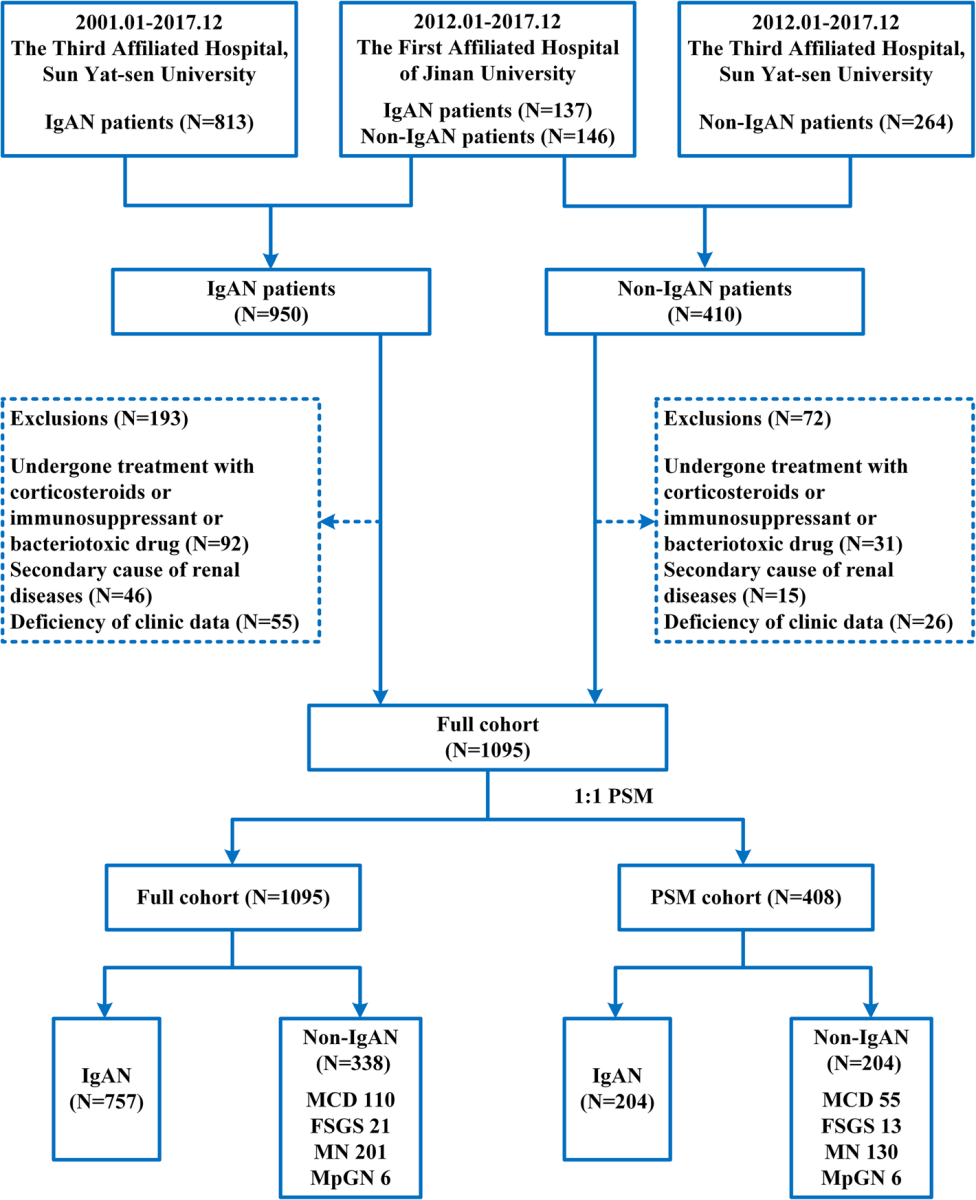
Study selection flow chart

Among IgAN patients, a total of 193 patients were excluded for the following reasons: 92 patients had undergone treatment with corticosteroids, immunosuppressants, or bacteriotoxic drugs; 46 patients had a secondary cause of IgAN; and 55 patients had a deficiency of clinical data. Among non-IgAN patients, 72 patients were excluded for the following reasons: 31 patients had undergone treatment with corticosteroids, immunosuppressants, or bacteriotoxic drugs; 15 patients had a secondary cause of renal disease; and 26 patients had a deficiency of clinical data (Fig. 1).

In total, 757 IgAN patients and 338 non-IgAN patients were enrolled in the study. In terms of causes of the non-IgAN primary renal diseases, 110 patients had minimal change disease (MCD), 21 patients had Focal Segmental Glomerular Sclerosis (FSGS), 201 patients had membranous nephropathy (MN), and 6 patients had mesangial proliferative glomerular nephritis (MpGN; Fig. 1).

One thousand ninety-five patients with primary glomerular nephropathy were assigned a 1:1 PSM using the PSM extension program in the SPSS software package. The PSM cohort included 204 IgAN patients and 204 non-IgAN patients. The composition of the non-IgAN group included 55 cases of MCD, 13 cases of FSGS, 20 cases of MN, and 6 cases of MpGN (Fig. 1).

### Measurements

#### Clinical data collection

We recorded demographic data including age, gender, course of disease, blood pressure (BP) and body mass index (BMI). Immunological parameters (serum IgG, IgM, IgA, C3, and C4 levels) were measured by turbidimetric immunoassays, and the reagents were adjusted according to the IFCC/CRM 470 certified reference material published by the International Federation of Clinical Chemistry. Proteinuria was tested by immunoturbidimetry, and urine samples were collected between 7 a.m. and 7 a.m. the following day to measure albuminuria levels over a 24 h period. Laboratory data [including cholesterol, triglyceride (TGs), high-density lipoprotein cholesterol (HDL-C), low-density lipoprotein cholesterol (LDL-C), hemoglobin, albumin, globulin, fasting glucose, uric acid, serum cystatin C, blood urea nitrogen (BUN), and serum creatinine (Scr)] were collected immediately upon patient admission. All the experimental indices were measured on a 7180 Biochemical Automatic Analyzer (Hitachi, Tokyo, Japan).

#### Renal assessment

According to data on Chinese chronic kidney disease (CKD) patients, the eGFR was calculated using a modified equation for Modification of Diet in Renal Disease (MDRD) [27]:

$$ \mathrm{eGFR}=175\times \mathrm{standardized}\ {\mathrm{Scr}}^{-1.234}\times {\mathrm{age}}^{-0.179}\times 0.79\ \left(\mathrm{if}\ \mathrm{female}\right) $$

$$ \left[\mathrm{Scr}\ \mathrm{is}\ \mathrm{measured}\ \mathrm{in}\ \mathrm{mg}/\mathrm{dL},\mathrm{and}\ \mathrm{age}\ \mathrm{in}\ \mathrm{years}\right] $$

#### Definitions

According to eGFR, CKD can be classified into 3 stages: 1) CKD1 stage (eGFR ≥90 mL/min per 1.73 m^2^), 2) CKD2 stage (eGFR 60–90 mL/min per 1.73 m^2^), and 3) CKD3–5stage (eGFR < 60 mL/min per 1.73 m^2^) [28]. In addition, patients were categorized into three groups according to proteinuria: ≤1 g/d, 1–3.5 g/d, and > 3.5 g/d [29]. According to the reagents tested in our hospital, serum IgA > 4.0 g/L was considered to be increased IgA and serum C3 < 0.8 g/L was considered to be decreased C3.

### Statistical analyses

In order to reduce the heterogeneity of data, IgAN cases were matched via PSM to non-IgAN cases on the logit of the propensity score with nearest neighbor matching in a 1:1 manner, with a caliper of 0.02 with no replacements, according to age, gender, BMI, proteinuria, and eGFR.

Continuous variables are presented as the mean ± standard deviation (SD), while non-parametric variables are presented as the median and interquartile ranges. Categorical variables are expressed as the frequency and percentage. The logarithmic transformation of proteinuria in regression analysis was performed because of the skewed distribution.

Student’s t-test, analysis of variance (ANOVA), or non-parametric test was used to compare continuous variables between groups, where appropriate. Differences between categorical variables were analyzed using a chi-square test or double-tailed Fisher’s exact test, depending on applicability.

In view of the absence of standardized cut-off points for serum IgG, IgA, IgM, C3, and C4 levels, as well as a cut-off for the IgA/C3 ratio, the diagnostic performance of IgAN was assessed using a receiver operating characteristic (ROC) curve, and the area under the ROC (AUROC) curve analysis, in order to choose the best cut-off point after maximizing for total sensitivity and specificity (maximum Youden index). A ROC curve was also drawn to examine the performance of the IgA/C3 ratio in predicting IgAN in every proteinuria classification, and in different CKD stages. Comparisons of the AUROC between groups were evaluated using a Z-test.

Multivariate logistic regression models were employed to study the association of indices of IgAN with age, gender, IgA/C3 ratio and other variables, with significant associations (*P* < 0.05) explored in a univariate logistic regression analysis.

All values are two-tailed, and *P* < 0.05 was considered statistically significant. Data were analyzed using IBM SPSS Statistics version 25.0 for Windows (IBM, Armonk, NY, USA) and Medcalc (Version 11.2, MedCalc Software, BE).

## Results

### Demographic and clinical characteristics of the study population

The mean age of the full patient cohort was 34.76 years, and 49.41% of the patient cohort was male. The median course of disease was 6 months, and the mean BP of patients was 142/90 mmHg. In the non-IgAN group, 48.82% of patients were classified as in CKD 1 stage, 34.02% as CKD2 stage, and 17.16% as CKD3–5stage. The prevalence of CKD in the IgAN group was 41.08% CKD1 stage, 30.78% CKD2 stage, and 28.14% CKD3–5stage (Table 1).

**Table 1:** Differences of demographic and clinical characteristics between IgAN patients and non-IgAN patients

	Full cohort				PSM cohort			
	non-IgAN (*N* = 338)	IgAN (*N* = 757)	Total (*N* = 1095)	*P*	non-IgAN (*N* = 204)	IgAN (N = 204)	Total (*N* = 408)	*P*
Age (years)	40.56 ± 16.48	32.17 ± 10.84	34.76 ± 13.42	< 0.001	42.96 ± 16.15	30.22 ± 11.18	36.59 ± 15.27	< 0.001
Male: female ratio	199:139	342:415	541:554	< 0.001	107:97	107:97	214:194	1.000
Course (months)	3 (1–12)	7 (2–14)	6 (1–24)	< 0.001	6 (1–18)	6 (1–24)	6 (1–22.5)	0.978
Clinic-SBP (mmHg)	132.95 ± 20.01	145.08 ± 26.42	141.64 ± 25.36	< 0.001	132.84 ± 20.28	141.74 ± 22.95	137.82 ± 22.23	< 0.001
Clinic-DBP (mmHg)	83.10 ± 12.38	92.66 ± 18.96	89.95 ± 17.87	< 0.001	83.00 ± 12.69	89.78 ± 16.11	86.79 ± 15.06	< 0.001
CKD stage				< 0.001				0.016
CKD1 (N/%)	165 (48.82%)	311 (41.08%)	476 (43.47%)		92 (45.10%)	98 (48.04%)	190 (46.57%)	
CKD2 (N/%)	115 (34.02%)	233 (30.78%)	348 (31.78%)		74 (36.27%)	50 (24.51%)	124 (30.39%)	
CKD3–5 (N/%)	58 (17.16%)	213 (28.14%)	271 (24.75%)		38 (18.63%)	56 (27.45%)	94 (23.04%)	
BMI (kg/m^2^)	23.64 ± 4.69	22.74 ± 3.78	23.23 ± 4.31	0.037	23.55 ± 4.96	22.47 ± 3.23	23.06 ± 4.29	0.055
Hematuria (N/%)	244 (72.19%)	592 (78.20%)	836 (76.35%)	0.031	145 (71.08%)	166 (81.37%)	311 (76.23%)	0.015
Proteinuria (g/d)	4.00 (2.17–6.90)	0.59 (0.22–1.50)	1.04 (0.34–3.46)	< 0.001	2.63 (1.21–4.06)	2.55 (0.75–4.38)	2.56 (0.96–4.21)	0.427
Proteinuria classifications				< 0.001				0.482
Proteinuria ≤1 g/d (N/%)	47 (13.91%)	495 (65.39%)	542 (49.50%)		47 (23.04%)	57 (27.94%)	104 (25.49%)	
Proteinuria 1–3.5 g/d (N/%)	99 (29.29%)	190 (25.10%)	289 (26.39%)		87 (42.65%)	78 (38.24%)	165 (40.44%)	
Proteinuria > 3.5 g/d (N/%)	192 (56.80%)	72 (9.51%)	264 (24.11%)		70 (34.31%)	69 (33.82%)	139 (34.07%)	
Hemoglobin (g/L)	130.93 ± 20.51	126.22 ± 22.02	127.69 ± 21.66	0.001	129.28 ± 19.39	125.85 ± 23.62	127.58 ± 21.63	0.112
Albumin (g/L)	24.80 ± 8.23	38.32 ± 7.43	34.12 ± 9.91	< 0.001	27.27 ± 8.88	33.92 ± 8.89	30.57 ± 9.48	< 0.001
Globulin (g/L)	21.13 ± 4.41	26.34 ± 4.86	24.71 ± 5.30	< 0.001	21.84 ± 4.42	24.08 ± 4.31	22.97 ± 4.50	< 0.001
fasting glucose (mmol/L)	5.07 ± 1.41	4.90 ± 0.96	4.99 ± 1.23	0.086	5.14 ± 1.49	4.84 ± 1.13	5.02 ± 1.37	0.051
Cholesterol (mmol/L)	8.61 ± 3.37	5.40 ± 2.14	6.40 ± 2.98	< 0.001	7.74 ± 3.38	6.41 ± 3.17	7.08 ± 3.34	< 0.001
Triglyceride (mmol/L)	2.41 ± 1.83	1.62 ± 1.37	1.87 ± 1.57	< 0.001	2.13 ± 1.43	1.96 ± 1.48	2.05 ± 1.46	0.257
HDL-C (mmol/L)	1.49 ± 0.50	1.30 ± 0.42	1.36 ± 0.45	< 0.001	1.48 ± 0.51	1.34 ± 0.44	1.41 ± 0.48	0.002
LDL-C (mmol/L)	5.69 ± 2.58	3.18 ± 1.53	4.01 ± 2.27	< 0.001	5.01 ± 2.51	3.98 ± 2.23	4.51 ± 2.43	< 0.001
Uric acid (mmol/L)	410.59 ± 120.67	422.00 ± 130.89	416.03 ± 125.68	0.254	408.26 ± 119.47	422.17 ± 138.14	413.75 ± 127.14	0.331
Serum Cystatin C (mg/L)	1.18 ± 0.66	1.42 ± 1.12	1.30 ± 0.92	0.002	1.23 ± 0.76	1.40 ± 1.13	1.30 ± 0.94	0.159
Blood urea nitrogen (mmol/L)	5.89 (4.40–9.27)	6.51 (4.84–10.01)	6.30 (4.57–9.56)	0.054	6.35 (4.54–9.56)	6.05 (4.58–9.31)	6.16 (4.56–9.52)	0.523
Serum creatinine (μmol/L)	76.00 (61.00–97.93)	80.50 (64.00–118.05)	78.70 (62.10–109.00)	0.002	76.60 (57.43–100.90)	78.00 (58.00–127.90)	77.65 (58.00–112.68)	0.196
eGFR-MDRD (ml/min/1.73m^2^)	90.28 ± 35.21	81.79 ± 38.50	84.43 ± 37.70	< 0.001	88.98 ± 38.83	91.36 ± 57.27	90.17 ± 48.88	0.624
IgG (g/L)	6.52 ± 3.55	10.58 ± 3.46	9.34 ± 3.96	< 0.001	7.34 ± 3.76	8.84 ± 3.74	8.09 ± 3.82	< 0.001
IgA (g/L)	2.13 ± 0.86	2.86 ± 1.11	2.64 ± 1.09	< 0.001	2.14 ± 0.85	2.84 ± 1.09	2.49 ± 1.03	< 0.001
IgM (g/L)	1.25 ± 0.65	1.44 ± 0.71	1.38 ± 0.70	< 0.001	1.24 ± 0.65	1.42 ± 0.73	1.33 ± 0.70	0.007
C3 (g/L)	1.15 ± 0.26	1.02 ± 0.26	1.06 ± 0.27	< 0.001	1.14 ± 0.26	1.05 ± 0.30	1.10 ± 0.28	0.001
C4 (g/L)	0.30 ± 0.11	0.26 ± 0.10	0.27 ± .011	< 0.001	0.30 ± 0.11	0.28 ± 0.11	0.29 ± 0.11	0.120
IgA/C3 ratio	1.94 ± 0.92	2.98 ± 1.27	2.66 ± 1.27	< 0.001	1.96 ± 0.91	2.87 ± 1.31	2.42 ± 1.22	< 0.001
increased IgA (N/%)	10 (2.96%)	97 (12.81%)	107 (9.77%)	< 0.001	5 (2.45%)	25 (12.25%)	30 (7.35%)	< 0.001
decreased C3 (N/%)	24 (7.10%)	143 (18.89%)	167 (15.25%)	< 0.001	14 (6.86%)	25 (12.25%)	39 (9.56%)	0.064
IgA/C3 ratio > 3.53 (N/%)	17 (5.03%)	196 (25.89%)	213 (19.45%)	< 0.001	10 (4.90%)	49 (24.02%)	59 (14.46%)	< 0.001

*BMI* body mass index, *C3* complement 3, *C4* complement 4, *CKD* chronic kidney disease, *DBP* diastolic blood pressure, *eGFR* estimated glomerular filtration rate, *HDL-C* high-density lipoprotein cholesterol, *IgA* immunoglobulin A, *IgAN* immunoglobulin A nephrology, *IgG* immunoglobulin G, *IgM* immunoglobulin M, *LDL-C* low-density lipoprotein cholesterol, *PSM* propensity score matched, *SBP* systolic blood pressure

*P* value for analysis of comparison between IgAN patients and non-IgAN patients

The median proteinuria in the IgAN group was 0.88 g/d, which was much lower than that of the non-IgAN group (4.00 g/d). 53.10% of IgAN patients had proteinuria ≤1 g/d, 33.55% had proteinuria between 1 and 3.5 g/d, and only 13.34% had proteinuria > 3.5 g/d. Among non-IgAN patients, the percentage of patients within the three proteinuria classifications were 13.91, 29.29, and 56.80% for proteinuria ≤1 g/d, proteinuria 1–3.5 g/d, and proteinuria > 3.5 g/d, respectively (Table 1).

Among the full cohort, compared to non-IgAN patients, patients with IgAN had a younger mean age, and were more likely to be female. They also had a longer disease course, a higher prevalence of hematuria and lower prevalence of proteinuria, and higher BP, serum albumin, serum globulin, serum cystatin C, BUN and Scr. IgAN patients also had lower BMIs, and lower levels of hemoglobin, cholesterol, triglycerides, HDL-C, LDL-C and eGFR (*P* < 0.05). IgAN patients had higher levels of IgG, IgA, IgM, IgA/C3 ratio and a higher prevalence of increased IgA, decreased C3 and IgA/C3 ratio > 3.53, as well as lower levels of C3 and C4 than non-IgAN patients (*P* < 0.05; Table 1).

After PSM, we pair-matched 204 IgAN patients with 204 non-IgAN patients, and found no differences in demographic and clinical characteristics, including gender, disease course, BMI, and proteinuria classification. There were also no differences in the levels of hemoglobin, serum fasting glucose, triglycerides, uric acid, serum cystatin C, BUN, Scr, eGFR and C4. In the PSM cohort, IgAN patients still had higher levels of IgG, IgA, IgM, IgA/C3 ratio and a higher prevalence of increased IgA and IgA/C3 ratio > 3.53, and lower C3 levels than non-IgAN patients (*P* < 0.05; Table 1).

### Comparison of the IgA/C3 ratio between IgAN and non-IgAN patients

In the full cohort and the PSM cohort, the mean serum IgA/C3 ratios of the IgAN groups were 2.98 ± 1.27 mmol/L and 2.87 ± 1.31 mmol/L, respectively, which were significantly higher than those of the non-IgAN groups (1.94 ± 0.92 mmol/L and 1.96 ± 0.91 mmol/L, respectively, both *P* < 0.001).

**Fig. 2:**
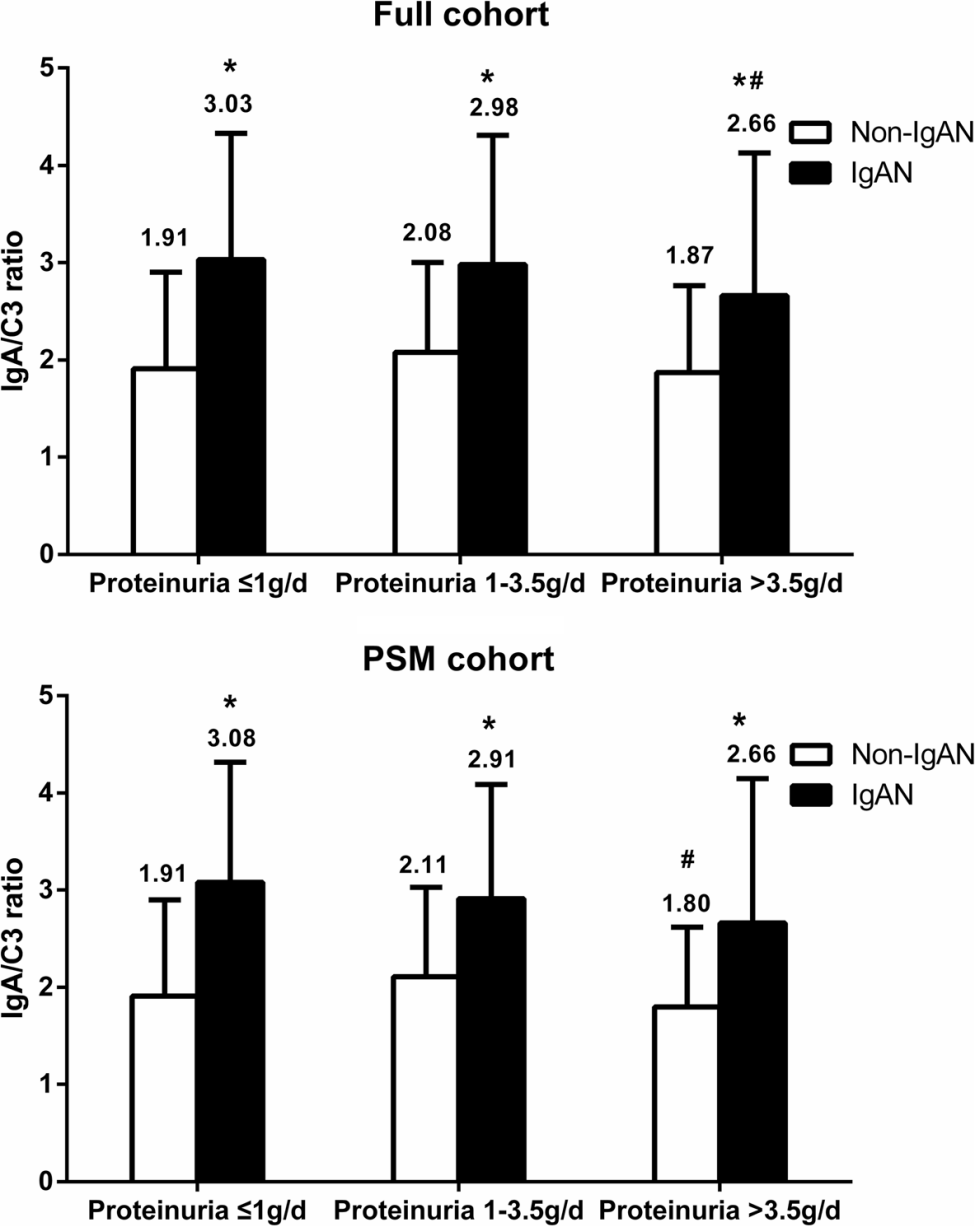
Comparison of the IgA/C3 ratio in different proteinuria levels. (* indicated comparison with Non-IgAN group, *p* < 0.05; # indicated comparison with proteinuria≤1 g/d group in the full cohort and comparison with proteinuria 1–3.5 g/d group in the PSM cohort, *p* < 0.05)

According to proteinuria levels, IgAN or non-IgAN patients were classified into three groups: ≤1 g/d, 1–3.5 g/d or > 3.5 g/d. IgAN patients in each cohort and in each of the three proteinuria groups had a higher IgA/C3 ratio than that of non-IgAN patients (all *P* < 0.001). Among the full cohort, only IgAN patients in the proteinuria> 3.5 g/d group had an IgA/C3 ratio lower than that of proteinuria≤1 g/d (*P* = 0.026), and non-IgAN patients in the proteinuria1–3.5 g/d group had an IgA/C3 ratio higher than that of proteinuria> 3.5 g/d in the PSM cohort (*P* = 0.026; Fig. 2).

**Fig. 3:**
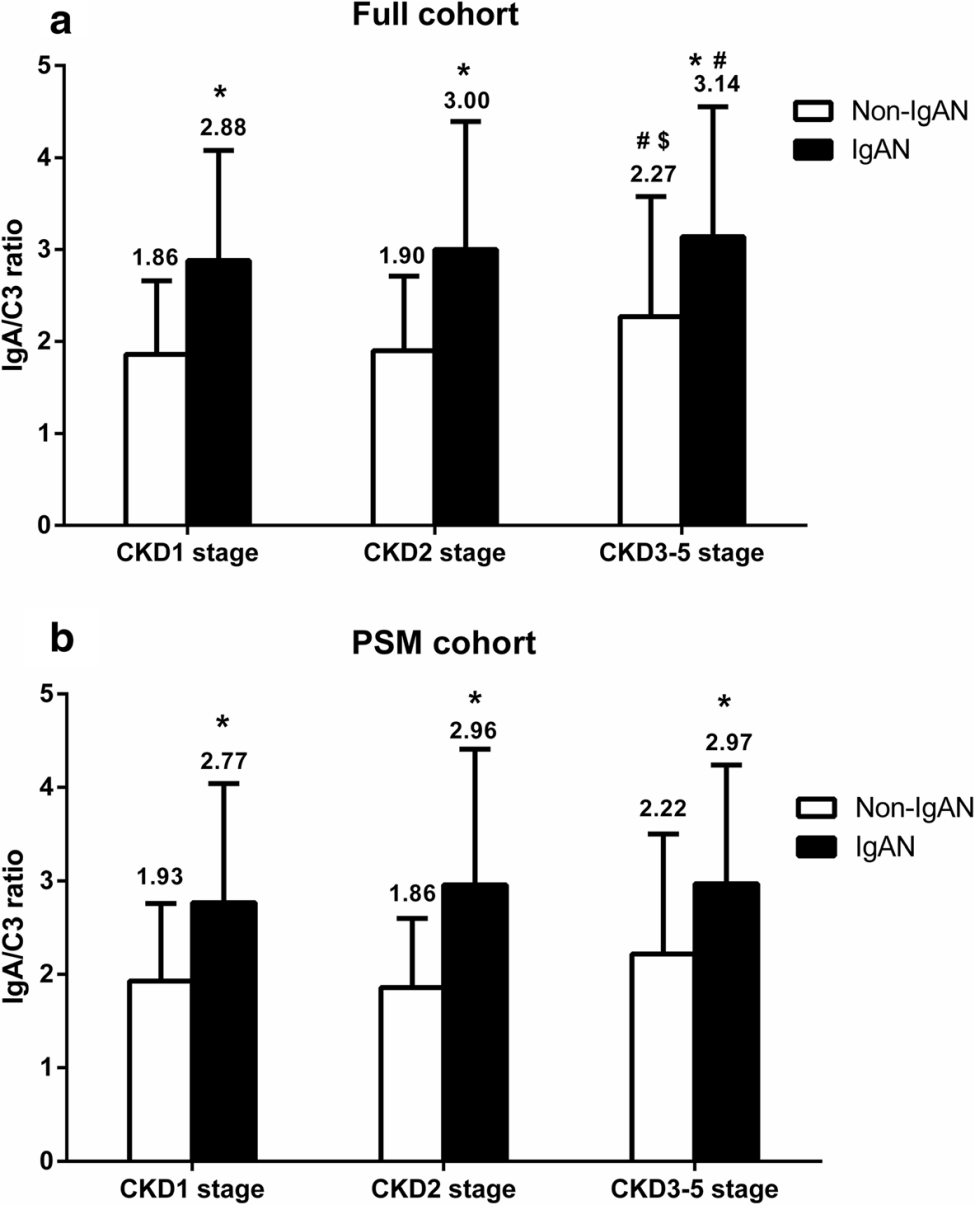
Comparison of the IgA/C3 ratio in different CKD stages. (* indicated comparison with Non-IgAN group, *p* < 0.05; # indicated comparison with CKD 1 stage, *p* < 0.05; $ indicated comparison with CKD 2 stage, *p* < 0.05)

We divided IgAN and non-IgAN patients into 3 CKD stages: CKD1, CKD2, and CKD3–5. In both the full cohort and the PSM cohort, the IgA/C3 ratio in patients with IgAN was significantly higher than that of non-IgAN patients, in all different groups (all *P* < 0.001). In the full cohort, non-IgAN patients in CKD3–5 stage had a higher IgA/C3 ratio than that of CKD1 and CKD2 stage patients (*P* = 0.005 and 0.021, respectively). IgAN patients in CKD3–5 stage had a higher IgA/C3 ratio than that of CKD1 stage patients (*P* = 0.023). There was no difference in the IgA/C3 ratio among non-IgAN and IgAN patients between different CKD groups in the PSM cohort (*P* = 0.116 and 0.578, respectively; Fig. 3).

### Predictors of IgAN

In order to distinguish between IgAN and non-IgAN, the ROC curve was used to assess the value of serum IgG, IgA, IgM, C3, and C4 levels, as well as the IgA/C3 ratio, in predicting IgAN. In both the full cohort and the PSM cohort, the AUROCs of the IgA/C3 ratio were as high as 0.767 (sensitivity 75.03%, specificity 67.46%, *P* < 0.001) and 0.734 (sensitivity 58.82%, specificity 77.45%, *P* < 0.001; Table 2 and Additional file 1: Figure S1).

**Table 2:** ROC curve of serum IgG, IgA, IgM, C3, C4 and IgA/C3 ratio in predicting IgAN

	Full cohort							PSM cohort				
	Area	*P*	Cut-off point	Sensitivity (%)	Specificity (%)	Youden index	Area	*P*	Cut-off point	Sensitivity (%)	Specificity (%)	Youden index
IgG	0.810	< 0.001	8.32	77.85	76.63	0.5448	0.629	< 0.001	8.32	58.13	68.63	0.2676
IgA	0.715	< 0.001	2.245	70.45	63.61	0.3415	0.696	< 0.001	2.12	74.51	56.37	0.3088
IgM	0.587	< 0.001	1.31	49.01	66.86	0.1587	0.580	0.005	1.20	54.90	61.76	0.1667
C3	0.657	< 0.001	1.081	66.84	60.06	0.2690	0.621	< 0.001	1.08	64.71	61.27	0.2598
C4	0.628	< 0.001	0.25	57.12	65.09	0.2221	0.558	0.044	0.25	49.50	63.24	0.1274
IgA/C3 ratio	0.767	< 0.001	2.102	75.03	67.46	0.4249	0.734	< 0.001	2.410	58.82	77.45	0.3627

*C3* complement 3, *C4* complement 4, *IgA* immunoglobulin A, *IgAN* immunoglobulin A nephrology, *IgG* immunoglobulin G, *IgM* immunoglobulin M, *PSM* propensity score matched, *ROC* receiver operating characteristic

In the different proteinuria groups, the highest AUROC of the IgA/C3 ratio in predicting IgAN was in the proteinuria≤1 g/d group (full cohort: AUROC 0.801, sensitivity 70.75%, specificity 80.85%; PSM cohort: AUROC 0.803, sensitivity 66.67%, specificity 82.98%; all *P* < 0.001), which was respectively significantly higher than that in the proteinuria> 3.5 g/d group (full cohort: AUROC 0.688, sensitivity 74.25%, specificity 59.16%; PSM cohort: AUROC 0.704, sensitivity 65.22%, specificity 70.00%; all *P* < 0.001) (full cohort: *P* = 0.011; PSM cohort: *P* = 0.021). There was no difference in the AUROC of the IgA/C3 ratio in predicting IgAN between all other groups (all *P* > 0.05; Table 3 and Additional file 2: Figure S2).

**Table 3:** ROC curve of IgA/C3 ratio in predicting IgAN in different proteinuria levels

	Full cohort							PSM cohort				
	Area	*P*	Cut-off point	Sensitivity (%)	Specificity (%)	Youden index	Area	*P*	Cut-off point	Sensitivity (%)	Specificity (%)	Youden index
Proteinuria ≤1 g/d	0.801	< 0.001	2.229	70.75	80.85	0.5160	0.803	< 0.001	2.352	66.67	82.98	0.4965
Proteinuria 1–3.5 g/d	0.726	< 0.001	2.458	64.32	73.47	0.3779	0.717	< 0.001	1.977	80.77	55.17	0.3594
Proteinuria > 3.5 g/d	0.688	< 0.001	1.833	74.29	59.16	0.3345	0.704	< 0.001	2.013	65.22	70.00	0.3522

*C3* complement 3, *IgA* immunoglobulin A, *IgAN* immunoglobulin A nephrology, *PSM* propensity score matched, *ROC* receiver operating characteristic

There was no difference in the AUROC of the IgA/C3 ratio in predicting IgAN between all the CKD groups (all *P* > 0.05; Table 4 and Additional file 3: Figure S3).

**Table 4:** ROC curve for serum IgA/C3 ratio in predicting IgAN in different renal function

	Full cohort							PSM cohort				
	Area	*P*	Cut-off point	Sensitivity (%)	Specificity (%)	Youden index	Area	*P*	Cut-off point	Sensitivity (%)	Specificity (%)	Youden index
CDK 1 stage	0.769	< 0.001	2.097	72.03	69.09	0.4112	0.719	< 0.001	2.578	54.08	81.52	0.3560
CKD 2 stage	0.788	< 0.001	2.229	72.10	74.68	0.4689	0.779	< 0.001	1.7521	92.00	51.35	0.4335
CKD 3–5 stage	0.703	< 0.001	1.977	84.98	56.90	0.4187	0.688	< 0.001	1.977	83.93	60.53	0.4445

*C3* complement 3, *CKD* chronic kidney disease, *IgA* immunoglobulin A, *IgAN* immunoglobulin A nephrology, *PSM* propensity score matched, *ROC* receiver operating characteristic

### Prediction efficiency of specific cut-off points of the IgA/C3 ratio

**Fig. 4:**
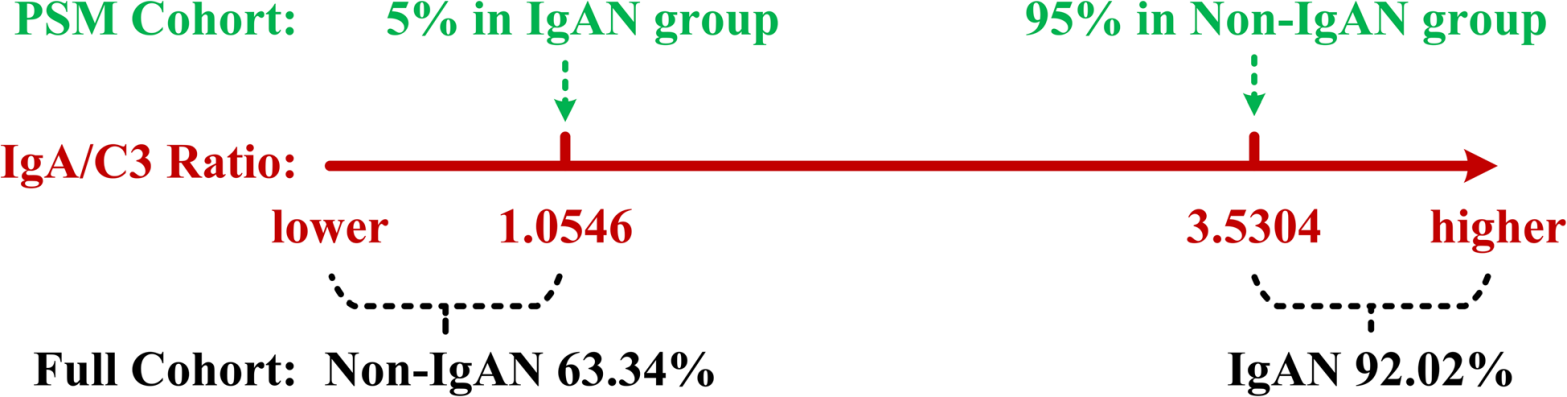
Prediction efficiency of specific cut-off points of the IgA/C3 ratio

In the PSM cohort, the 95% cut-off point for the IgA/C3 ratio in the non-IgAN group was 3.5304, and the 5% cut-off point for the IgA/C3 ratio in the IgAN group was 1.0546. This cut-off assumes that patients with an IgA/C3 ratio > 3.5304 are predicted to be IgAN, and patients with an IgA/C3 ratio < 1.0546 are non-IgAN. In the full cohort, the diagnostic accordance rate of an IgAN diagnosis among all patients with an IgA/C3 ratio > 3.5304 was as high as 92.02%, but the diagnostic accordance rate of a diagnosis of non-IgAN among all patients with an IgA/C3 ratio < 1.0546 was only 63.34% (Fig. 4).

### Factors associated with IgAN

In the full cohort, a multivariate logistic regression analysis was performed to clarify which factors were independently associated with IgAN. The analysis showed that the IgA/C3 ratio was independently correlated with IgAN, in multivariate adjusted models (all *P* < 0.001; Table 5).

**Table 5:** Multivariate logistic regression analysis for IgAN (1 = non-IgAN; 2 = IgAN) in the full cohort

	OR (95% CI)	*P* Value
Model 1 R^2^ = 0.233		
IgA/C3 ratio (per 1)	2.676 (2.268–3.158)	< 0.001
Model 2 R^2^ = 0.229		
Age (per 1 year)	0.952 (0.941–0.962)	< 0.001
IgA/C3 ratio (per 1)	2.742 (2.307–3.260)	< 0.001
Model 3 R^2^ = 0.360		
Age (per 1 year)	0.936 (0.923–0.948)	< 0.001
IgG (per 1 mmol/L)	1.385 (1.315–1.458)	< 0.001
IgA/C3 ratio (per 1)	2.051 (1.702–2.471)	< 0.001
Model 4 R^2^ = 0.466		
Age (per 1 year)	0.932 (0.918–0.947)	< 0.001
Albumin (per 1 g/L)	1.198 (1.171–1.226)	< 0.001
CKD stage		
CKD2 (versus CKD1)	2.278 (1.407–3.687)	0.001
CKD3–5 (versus CKD1)	4.782 (2.766–8.269)	< 0.001
IgA/C3 ratio (per 1)	2.159 (1.759–2.650)	< 0.001

Model 1: IgA/C3 ratio, univariate

Model 2: IgA/C3 ratio, age and gender (male = 1; female = 2) adjusted

Model 3: IgA/C3 ratio, age, gender (male = 1; female = 2), IgG, IgA, IgM, C3, C4 adjusted

Model 4: multivariable adjusted

Variables of univariate regression analysis include age, gender (male = 1, female = 2), course, clinic-SBP, clinic-DBP, BMI, hematuria, Lg (proteinuria), proteinuria stage, hemoglobin, albumin, uric acid, serum cystatin, blood urea nitrogen, serum creatinine, eGFR-MDRD, CKD stage, IgG, IgA, IgM, C3, C4 and IgA/C3 ratio

All variables with significant associations in univariate regression analysis were included in Model 4 multivariate regression analysis

*BMI* body mass index, *C3* complement 3, *C4* complement 4, *CI* confidence interval, *CKD* chronic kidney disease, *DBP* diastolic blood pressure, *eGFR* estimated glomerular filtration rate, *HDL-C* high-density lipoprotein cholesterol, *IgA* immunoglobulin A, *IgAN* immunoglobulin A nephrology, *IgG* immunoglobulin G, *IgM* immunoglobulin M, *LDL-C* low-density lipoprotein cholesterol, *OR* odds ratio, *PSM* propensity score matched, *SBP* systolic blood pressure

## Discussion

In the present study, we assessed 1095 primary glomerular nephropathy patients, including 757 IgAN patients and 338 Non-IgAN patients, to investigate whether a high IgA/C3 ratio could be used as a diagnostic criterion for IgAN, using a PSM method. Our study revealed that both in the full cohort and PSM cohort, the serum IgA/C3 ratio of the IgAN was significantly higher than that of the non-IgAN. The same results were also obtained when comparisons were stratified by level of proteinuria or level of renal function. In the PSM cohort, there was no difference in the IgA/C3 ratio of IgAN patients between different proteinuria groups and different CKD stages. The AUROC for the IgA/C3 ratio was 0.767 in the full cohort, and 0.734 in the PSM cohort in distinguishing IgAN among primary glomerular disease. The highest AUROC of the IgA/C3 ratio for an IgAN diagnosis was in the proteinuria≤1 g/d group (0.801 in the full cohort, and 0.803 in the PSM cohort); however, there was no difference in the AUROC of the IgA/C3 ratio between all CKD groups. Meanwhile, the diagnostic accordance rate for a diagnosis of IgAN among all patients with an IgA/C3 ratio > 3.5304 was as high as 92.02% in the full cohort. A multivariate logistic regression analysis showed that IgAN was independently correlated with IgA/C3 ratio.

In recent years, studies have confirmed that IgAN is a complex immune disease that involves deposits of the circulating immune complexes (CIC) IgA, C3, and IgG in the mesangial region of the glomeruli [11]. Although the pathophysiology of IgAN is still unclear, researchers currently favor a four-hit hypothesis, wherein GdIgA1 plays a key pathogenic role [11, 12]. Firstly, increased levels of circulating GdIgA1 with aberrant O-glycosylation of its hinge region result in exposed N-acetylgalactosamine. And then, N-acetylgalactosamine results in the production of unique antiglycan antibodies Gd-IgA1-binding proteins, which are thought to target the terminal N-acetylgalactosamine in the hinge region of Gd-IgA1. The third hit is the formation of pathogenic Gd-IgA1–containing CICs. Finally, these complexes are deposited in the mesangial region, leading to glomerular injury [11, 12, 30]. Studies have also revealed that mesangial IgA can activate C3, which speeds the production of terminal complex c5b-9, which in-turn activates macrophage to produce inflammatory mediators and matrix proteins. Thus, both IgA and C3 play an important role in the development and occurrence of IgAN. Due to glomerular deposition of IgA1 or IgA1-IgG immune complexes, serum C3 levels are lower in IgAN, and together with elevated serum IgA, this result in a higher IgA/C3 ratio, which can be used as a biomarker for IgAN [31]. In addition to measurements of pure serum IgA or C3 levels, previous studies have also found that the serum IgA/C3 ratio can clearly distinguish between severe IgA histological lesions and mild IgA histological lesions [13, 22, 32]. Our study is in line with these previous results, as in our PSM cohort, the AUROC of the IgA/C3 ratio for the diagnosis of IgAN reached 0.734, while the AUROC of IgA and C3 alone were only 0.696 and 0.621, respectively. However, additional data from larger samples are still needed, especially as previous studies have all examined relatively small patient populations.

In the present study, IgAN patients had a significantly higher IgA/C3 ratio than that of non-IgAN primary glomerular disease patients, in both the full cohort and the PSM cohort. Our results are consistent with previous studies [13, 14, 21, 22], and are also the first to demonstrate the predictive value of IgA/C3 ratio among primary glomerular nephropathy patients between different levels of proteinuria and different renal functions. The cut-off value of the serum IgA/C3 ratio was 2.102 in the full cohort, with an AUROC as high as 0.767 and 2.410, and an AUROC as high as 0.734 in the PSM cohort. These results are close to the cut-off value of 2.14 with an AUROC of 0.71, which was reported by Yasuhiko T et al. in a comparison of IgAN and non-IgAN patients [13]. The Yasuhiko T team also reported that the serum IgA/C3 ratio might be used to diagnose and predict prognostic grading in patients with IgAN [33], and combined with microscopic hematuria and/or persistent proteinuria, high serum IgA levels, and the serum IgA/C3 ratio could be used to distinguish IgAN from other primary renal diseases [34]. A study that collected data from 44 children with IgAN treated with multi-drug combination therapy showed that combined serum IgA/C3 ratio and glomerular C3 staining could predict the prognosis of IgA nephropathy [35]. However, a study involving 1564 IgAN patients indicated that a decrease in serum C3 levels in IgAN was not associated with renal progression [36].

Persistent proteinuria has proven to be an adverse prognostic factor for IgAN, as its progression varies with different levels of proteinuria. A follow-up study by Heather N. R et al. showed that when proteinuria level was < 1 g/d, the renal function of IgAN patients decreased at a 90% slower rate than average. The rate of renal function decrease was accelerated with increased proteinuria, and in IgAN patients with continuous proteinuria > 3 g/d, the decrease in renal function was 25 times faster than that of patients with proteinuria < 1 g/d [29]. Studies have also shown that compared to other types of progressive glomerulonephritis, IgAN appears to be associated with a poorer prognosis when presenting with nephrotic proteinuria between 3 and 3.5 g/d. Therefore, it has been suggested that IgAN with proteinuria < 1 g/d often indicates a favorable prognosis [29, 37–40]. Based on these observations, we divided proteinuria into three levels: ≤1 g/d, 1–3 g/d and > 3.5 g/d, and revealed that patients with proteinuria ≤1 g/d had the highest AUROC of the IgA/C3 ratio (which was higher than 0.80 in both the full cohort and the PSM cohort with cut-off points 2.229 and 2.352, respectively) in distinguishing IgAN among primary glomerular disease, while those in the proteinuria > 3.5 g/d group had the lowest. Meanwhile, renal function was also divided into three groups (eGFR ≥90, 90–60, and < 60 mL/min per 1.73 m^2^), but we found no difference in the AUROC of the IgA/C3 ratio for the diagnosis of IgAN between these groups.

In China, there are many nephropathy patients without access to renal biopsy. This is for a variety of reasons – such as the uneven distribution of medical resources, the prevalence of rural hospitals without access to biopsy technology, incidences of renal function impaired to a level unable to tolerate renal biopsy, and a prevalence of patients with a low level of proteinuria (i.e. < 1 g/d) with no obvious clinical symptoms, who elect not to undergo this invasive procedure. However, the prevalence of IgAN is very high in China, and therefore there is a strong need for noninvasive, readily available clinical indicators that better predict IgAN. Our study has shown that the IgA/C3 ratio, a non-invasive index, is of high predictive value for IgAN, especially in patients with primary glomerulonephritis with proteinuria < 1 g/d. Moreover, the predictive value of the IgA/C3 ratio is not affected by kidney function.

In analyzing non-experimental data without a random distribution, differences between groups must be considered, and the PSM approach simplifies this problem by considering only one dimension: the propensity score dimension. Rather than matching all variables, you may compare individual subjects based only on their propensity score [23–26, 41]. As a novel and effective statistical tool that is increasingly being used in clinical studies, the PSM method is able to correct initial data mismatch between control groups. In the present study, there were significant differences between the two groups in baseline indicators such as age, gender, proteinuria, BMI, and eGFR prior to matching. In order to exclude the influence of confounders, we used the PSM method to balance these non-observational factors to reach more reliable conclusions. In the end, we achieved very similar results in both the full cohort and the PSM cohort, indicating that the clinical significance of these indicators is trustworthy. Using this improved statistical method, we calculated the 95% cut-off point for the IgA/C3 ratio in the non-IgAN population, which was 3.5304, and the diagnostic accordance rate of an IgAN diagnosis among all patients with an IgA/C3 ratio > 3.5304 was as high as 92.02% in the full cohort. Moreover, by multivariate logistic regression analysis of the full cohort, we demonstrated that IgAN was independently correlated with the IgA/C3 ratio. Together this analysis demonstrates that our measured indicators are consistent with observed clinical symptoms.

The present study has several advantages. Firstly, we used a large sample size to prove that the IgA/C3 ratio is of diagnostic value to IgAN. Secondly, a stratified analysis was carried out, controlling for different levels of renal function and levels of proteinuria. The results showed that the IgA/C3 ratio, as a non-invasive index, was of higher predictive value for IgAN in patients with primary glomerulonephritis, particularly those whose proteinuria was less than 1 g/d, and was not affected by renal function. Thirdly, we suggest that this index can be applicable to patients with glomerulonephropathy in remote areas without access to renal biopsy, and to patients with severe renal failure where there is an inability to perform renal biopsy. The limitations to our study include a relatively small number of non-IgAN patients, particular those with normal GFR and proteinuria≤1 g/d, the inclusion of only two units of data, lack of the correlation between of IgA/C3 ratio and histopatological MEST score and the lack of multicenter and follow-up data. Additionally, as a cross-sectional clinical study, we were unable to explain why the IgA/C3 ratio has higher diagnostic efficacy in patients with proteinuria < 1 g/d and the potential role of IgA/C3 ratio as prognostic marker of therapeutic response. Finally, as an observational study, we cannot firmly establish a cause and effect relationship between IgA/C3 ratio and IgAN, thus additional basic research is needed.

## Conclusions

In conclusion, the results of the present study provide clear evidence that the IgA/C3 ratio is a valid predictor for diagnosis of IgAN, particularly in patients with proteinuria less than 1 g/d. Additional multicenter large-scale studies are necessary in order to investigate the validity of this biomarker, and determine a standardized cut-off value.

## Additional files

Additional file 1: **Figure S1.** ROC curve of serum IgG, IgA, IgM, C3, C4 and IgA/C3 ratio in predicting IgAN. (TIF 1613 kb)

Additional file 2: **Figure S2.** ROC curve of IgA/C3 ratio in predicting IgAN in different proteinuria levels. (TIF 3214 kb)

Additional file 3: **Figure S3.** ROC curve of IgA/C3 ratio in predicting IgAN in different CKD stages. (TIF 3265 kb)
